# Diverse Attitudes and Experiences With Technology Use During the COVID-19 Pandemic Among Asian American and Pacific Islander Adults (the COMPASS Study): Survey Study

**DOI:** 10.2196/64999

**Published:** 2025-09-10

**Authors:** Linda G Park, Serena Chi, Myka Lay, Nicole Phan, Janice Y Tsoh, Oanh L Meyer, Bora Nam, Van Ta Park

**Affiliations:** 1 Department of Community Health Systems University of California, San Francisco School of Nursing San Francisco, CA United States; 2 School of Medicine Yale University New Haven, CT United States; 3 Samuel Merritt University Oakland, CA United States; 4 Department of Psychiatry and Behavioral Sciences University of California, San Francisco School of Medicine San Francisco, CA United States; 5 Asian American Research Center on Health University of California, San Francisco San Francisco, CA United States; 6 Multiethnic Health Equity Research Center University of California, San Francisco San Francisco, CA United States; 7 Department of Neurology University of California, Davis School of Medicine Sacramento, CA United States

**Keywords:** COVID-19, technology use, Asian American and Pacific Islander, mental health, physical health, social connections, virtual space, health disparities, thematic analysis, community partners, social media, multilingual survey, qualitative research, SARS-COV-2, coronavirus, infectious, pandemic, Asian American, Pacific Islander, mixed methods, COVID-19 Effects on the Mental and Physical Health of Asian Americans and Pacific Islanders Survey Study, COMPASS, adult, mobile health, mHealth

## Abstract

**Background:**

The COVID-19 pandemic forced the world to quarantine to slow the rate of transmission, causing communities to transition into virtual spaces. Asian American and Pacific Islander communities faced the additional challenge of discrimination that stemmed from racist and xenophobic rhetoric in the media. Limited data exist on technology use among Asian American and Pacific Islander adults during the height of the COVID-19 shelter-in-place period and its effect on their physical and mental health.

**Objective:**

This study aims to examine Asian American and Pacific Islander adults’ attitudes, perspectives, and experiences regarding their use of technology during the COVID-19 pandemic.

**Methods:**

We collaborated with community partners and used social media to distribute the COVID-19 Effects on the Mental and Physical Health of Asian Americans and Pacific Islanders Survey Study, a nationwide multilingual survey available in English, Chinese, Korean, Samoan, and Vietnamese. The survey was administered from October 2020 to February 2021, and participants rated their level of agreement (1=*not at all* to 5=*extremely*) on 6 items assessing their attitudes toward technology use. Thematic analysis was conducted on responses to the open-ended question “Is there anything else you want to tell us about your use of technology during COVID-19?” The qualitative responses were reviewed, analyzed, coded, and organized into corresponding themes.

**Results:**

The mean age of respondents was 45.9 (SD 16.3; range 18-98) years, with 5398 participants completing the quantitative survey and 1115 (20.66%) providing unique responses to the open-ended question. In the quantitative survey, 68% (3671/5398) of the respondents reported being comfortable using technology; the majority indicated that it helped them keep up with the news (4318/5398, 79.99%), maintain social connections (4102/5398, 75.99%), and provide care for others (2537/5398, 46.99%). However, responses were mixed regarding the usefulness of technology for health: 39.99% (2159/5398) agreed that it was helpful for mental health but disagreed regarding physical health. Four main themes emerged from the qualitative analysis: (1) technology was critical for functioning across many aspects of life and maintaining physical, mental, and emotional well-being; (2) technology was often the only means of interpersonal social connections; (3) overuse led to negative physical and mental health outcomes; and (4) technology use was associated with multiple challenges and barriers.

**Conclusions:**

Our findings revealed diverse perspectives and experiences related to technology use by Asian American and Pacific Islander adults during the height of the COVID-19 pandemic. Dependence on technology may have exacerbated social inequities, particularly for those with lack of access to devices and Wi-Fi and limited English proficiency, affecting their ability to work, apply for jobs, and communicate virtually. Further qualitative research would be beneficial in amplifying the perspectives of Asian American and Pacific Islander adults to uncover concerns and address health disparities.

## Introduction

### Background

The impact of technology on society in the realm of health care access and delivery has been profound and multifaceted. The dependence on technology was magnified during the COVID-19 pandemic, underscoring the critical role that technology plays in nearly every form of communication. The pandemic caused widespread loss of life, a myriad of health consequences, and unprecedented physical and social isolation [1]. During this period, technology served as a vital tool for accessing health care, continuing remote work and education, and operating businesses. To protect older adults and others considered vulnerable, shelter-in-place policies were implemented to varying degrees across the world. As a result, many were forced to rely on virtual forms of communication, such as videoconferencing and online chat. In most cases, virtual communication became the only means of social interaction.

The pandemic also brought the digital divide to the forefront. Disparities related to COVID-19 and health care access were exacerbated for rural communities, racial and ethnic minority groups, individuals with low income, and other populations considered vulnerable. These groups were disproportionately burdened by the digital divide, compounding existing structural disadvantages [2]. These inequities may have been particularly acute for individuals with limited English proficiency in virtual workspaces and job interviews, where reliance on verbal communication can heighten communication challenges [3]. Research has also revealed that the widening of the digital divide intensified challenges, especially for rural residents [4]. However, to our knowledge, limited research exists on the use of digital technology among Asian American and Pacific Islander populations in the United States during the pandemic, particularly among older adults (aged ≥60 y) and adults with limited English proficiency. Asian American and Pacific Islander populations were especially vulnerable during the pandemic, as xenophobic attitudes became more prevalent due to racist statements linking China to COVID-19 [5]. As a result, many individuals from these communities experienced racism, discrimination, and hate crimes, which contributed to fear, loneliness, and social isolation [6,7].

A Pew Research Center survey of American adults conducted in April 2021 revealed that the internet served as a “lifeline” for many during the pandemic, but respondents also reported “struggles” associated with the increased use of and reliance on the internet [8]. Such surveys provide insights into general patterns of digital technology use, purposes, and associated challenges among the general American adult population. However, limited data exist on digital technology use patterns, perceived health-related support, and attitudes toward digital technology use among Asian American and Pacific Islander adults. Examining these gaps may assist in culturally and linguistically tailoring appropriate digital technology programs for these diverse populations. In addition, a better understanding of the impact of digital technology use is needed to inform clinical recommendations for physical and mental health.

Our prior work with the COVID-19 Effects on the Mental and Physical Health of Asian Americans and Pacific Islanders Survey Study (COMPASS) revealed that increased technology use during the pandemic was associated with poorer mental and physical health outcomes among participants [9]. Specifically, those who reported increasing their technology use by ≥7 hours per day experienced higher levels of depressive symptoms and worse physical health, regardless of age or ethnic background [9]. These findings suggest that even with high levels of digital access, increased technology use does not necessarily equate to positive health outcomes. Still, little is known about digital technology use among Asian American and Pacific Islander adults. In the COMPASS study, the majority of Asian American and Pacific Islander participants reported owning a smartphone (96.2%), home internet access (90%), a desktop or laptop computer (83.4%), a tablet (61.1%), cable television (43.5%), and a streaming device (45.7%).

### Objectives

Building on previous findings, we aimed to address gaps in knowledge regarding technology use among Asian American and Pacific Islander populations by analyzing data from COMPASS, which examined mental and physical health implications associated with technology use during the height of the COVID-19 shelter-in-place period among Asian American and Pacific Islander adults in the United States. In this study, we quantitatively describe COMPASS participants’ attitudes toward technology use and qualitatively explore their perspectives and experiences of technology use during the COVID-19 pandemic.

## Methods

### Recruitment

A total of 5411 individuals participated in the study from October 2020 to February 2021. The survey was offered on the COMPASS website [10] in English, simplified Chinese, traditional Chinese, Korean, Vietnamese, and Samoan or by telephone with assistance from COMPASS staff and community partners (Multimedia Appendix 1). The survey was also offered by telephone in Cantonese, Mandarin, Vietnamese, Korean, and Samoan by multilingual COMPASS staff. The survey was shared through personal and professional networks via social media, email, telephone, health promotion webinars, flyers, and ethnic media across the United States. The 26 COMPASS community advisory board members and 15 community partners that serve Asian American and Pacific Islander populations supported recruitment efforts by promoting the survey among their networks. In addition, recruitment leveraged the Collaborative Approach for Asian Americans and Pacific Islanders Research and Education (CARE) registry [11,12], contacting eligible participants interested in participating in health research via email and telephone. The goal of the CARE registry was to increase the participation of Asian American and Pacific Islander individuals in research related to aging, Alzheimer disease and related dementias, and caregiving.

### Ethical Considerations

The COMPASS research project was approved by the institutional review board of the University of California San Francisco (UCSF; 20-31925). Informed consent was provided by participants before survey completion. Participants had the option to receive a US $10 gift card upon completing the survey.

### Eligibility and Procedures

Eligible participants were required to be aged ≥18 years; reside in the United States; and identify as Asian, Asian American, Native Hawaiian, Pacific Islander, or any combination of these with other racial or ethnic identities. Participants were also required to be able to read in English, Chinese (simplified or traditional), Korean, Samoan, or Vietnamese. We used the World Health Organization’s instrument adaptation process [13], including backward and forward translation, to guide the multilanguage survey’s scale and material development. All translated instruments were reviewed by multiple research team members for accuracy and cultural relevance. Non-English survey responses were translated into English through online translation services before analysis.

### Methodology, Data Collection, and Measures

We applied a cross-sectional study design that included COMPASS quantitative survey data and qualitative data from an open-ended question asking, “Is there anything else you want to tell us about your use of technology during COVID-19?” Free-text responses of any length were accepted. The data were stored in REDCap (Research Electronic Data Capture; Vanderbilt University) tools hosted at UCSF [14,15]. For sociodemographic information, participants were queried about their age, sex, ethnic or cultural background, marital status, country of birth, education, employment status, annual household income, and English proficiency.

The technology use survey was developed by the COMPASS research team to explore patterns of technology use during the COVID-19 pandemic. Participants were queried about the number of hours they used technology per day and whether their technology use increased during the COVID-19 crisis. Responses were categorized into five groups: (1) “did not increase,” (2) “increased by 1 to 2 hours per day,” (3) “increased by 3 to 4 hours per day,” (4) “increased by 5 to 6 hours per day,” and (5) “increased by ≥7 hours per day.” In addition, participants were asked about their comfort level with using technology and their engagement in various technology-mediated activities during the COVID-19 pandemic. These activities included video chatting with friends or family, video meetings for work-related activities, telephone or video visits with health care providers, exercise or physical fitness activities, mental health activities, keeping in touch with friends and family through social media, leisure activities or hobbies, and accessing news. Moreover, participants were asked to evaluate the helpfulness of technology. The survey encompassed statements concerning the use of technology for keeping up with the news, maintaining social connections, caregiving, supporting mental health, and enhancing physical health. Respondents indicated their level of agreement with each statement on a 5-point Likert scale ranging from 1=*not at all* to 5=*extremely*.

### Analysis

Descriptive statistics were used to report the sociodemographic characteristics and technology use patterns of the sample. Qualitative analysis was conducted using content analysis. Two trained independent reviewers initially organized the raw participant responses and analyzed the data to identify subthemes and categorize significant statements. Using an iterative process and a constant comparative method, the research team refined the coding scheme, finalized the themes, and identified patterns and relationships across the qualitative data.

Coding and organization of interview data were conducted using Microsoft Word and Microsoft Excel. A third coder independently cross-checked the transcripts to ensure the consistency of the interpretation and summarized the coded content within thematic categories. After this initial round of analysis, the coders collaboratively reviewed subthemes and worked together to consolidate them under broader thematic umbrellas. Through continued discussion, 5 major themes were identified. Codes were developed to support each theme, with refined subthemes providing additional structure. Participant responses were then organized according to these codes to illustrate and support the thematic findings.

The first coder (ML) is a board-certified registered nurse and research analyst at UCSF, with extensive experience in public health, clinical nursing, and Asian American and Pacific Islander health promotion initiatives. Their professional background spans diverse health care settings, with a focus on community health; health equity; and holistic, family-centered care. The second coder (NP) is a project policy analyst at UCSF with a strong background in qualitative research and experience engaging with research participants in both English and languages across several Asian American and Pacific Islander communities. Their expertise includes community-based participatory research, coordinating outreach initiatives, and developing volunteer programs for Asian American and Pacific Islander students. A third team member (LGP), also experienced in qualitative methods, served as an adjudicator to resolve discrepancies in data interpretation and assist in finalizing the thematic structure. The diverse backgrounds of all three coders informed their analytical lens, particularly in identifying sociocultural influences and health disparities within participants’ narratives. Their lived experiences and cultural identities contributed to a reflexive and culturally responsive approach to qualitative analysis.

## Results

### Participant Characteristics

Only individuals who provided responses to the open-ended question (n=1115) were included in the analysis. The mean age of the participants was 45.9 (SD 16.3; range 18-98) years. The majority identified as Vietnamese (399/1115, 35.78%) and ethnic Chinese (336/1115, 30.13%), followed by those who identified as Korean (161/1115, 14.44%) and Filipino (69/1115, 6.19%). Most of the participants completed the qualitative question in the survey in English (665/1115, 59.64%), identified as female (731/1115, 65.56%), were either married or living with a partner (723/1115, 64.84%), and were born in a foreign country (776/1115, 69.6%). In addition, a significant portion of participants reported limited English proficiency (322/1115, 28.88%). Table 1 provides an overview of the demographic characteristics of the participants.

**Table 1:** Descriptive characteristics of the participants (n=1115).

Characteristics			Participants
Age (y), mean (SD; range)			45.9 (16.3; 18-98)
**Age groups (y), n (%)**			
	<30	185 (16.6)	
	30-39	150 (13.4)	
	40-49	198 (17.8)	
	50-59	227 (20.4)	
	≥60	355 (31.8)	
**Questionnaire asked for cultural group, n (%)**			
	Asian Indian	68 (6.1)	
	Ethnic Chinese^a^	336 (30.1)	
	Filipino	69 (6.2)	
	Hmong	98 (2.1)	
	Japanese	61 (5.5)	
	Korean	161 (14.4)	
	Native Hawaiian or Pacific Islander	15 (1.3)	
	Vietnamese	399 (35.8)	
	Other or mixed	83 (7.5)	
**Survey response language, n (%)**			
	English	665 (59.6)	
	Chinese	78 (7)	
	Korean	81 (7.3)	
	Vietnamese	288 (25.8)	
	Samoan	3 (0.3)	
**Sex, n (%)**			
	Female	731 (65.6)	
	Male	380 (34.1)	
	Other or declined to answer	40 (0.9)	
**Country of birth, n (%)**			
	United States	338 (30.3)	
	Outside the United States	776 (69.6)	
	Don’t know	1 (0.1)	
**Limited English proficiency, n (%)**			
	Yes	322 (28.9)	
	No	793 (71.1)	
**Marital status, n (%)**			
	Single	275 (24.7)	
	Married or living with partner	723 (64.8)	
	Separated, divorced, or widowed	113 (10.1)	
	Declined to answer	4 (0.4)	
**Employment status, n (%)**			
	Full time	441 (39.6)	
	Part time	185 (16.6)	
	Homemaker	108 (9.7)	
	Unemployed	134 (12)	
	Retired	207 (18.6)	
	Other or declined to answer	82 (7.4)	
**Education, n (%)**			
	High school or less	246 (22.1)	
	Some college or technical school	162 (14.5)	
	Bachelor’s degree	379 (34)	
	Master’s degree or higher	318 (28.5)	
	Declined to answer	10 (0.9)	
**Household income (US $), n (%)**			
	≤25,000	282 (25.3)	
	>25,000-75,000	337 (30.2)	
	>75,000-150,000	232 (20.8)	
	>150,000	77 (6.9)	
	Declined to state	97 (8.7)	

^a^Ethnic Chinese includes individuals who identified as mainland Chinese, Hong Kongers, Taiwanese, or Huaren.

The participants’ technology use characteristics are presented in Table 2. The majority (644/1115, 57.8%) reported using technology for 1 to 8 hours per day. A notable portion (803/1115, 72%) reported increased technology use due to the COVID-19 pandemic. More than half of the respondents (627/1115, 56.23%) said that they were very or extremely comfortable using technology. During the pandemic, the most common activity was video chatting with friends or family, with 902 (81%) of the 1115 participants using platforms such as Zoom, WhatsApp, or FaceTime for communication. In addition, 614 (55.07%) participants engaged in video meetings for work-related activities, while 575 (51.57%) used technology for telephone or video visits with health care providers. Exercise or physical fitness activities were performed by 420 (37.67%) of the 1115 participants, while 187 (16.77%) reported engaging in mental health activities through technology. Social media served as a means of keeping in touch with friends and family for 866 (77.67%) of the 1115 participants, and 517 (46.37%) used technology for leisure activities or hobbies. Furthermore, 850 (76.23%) participants accessed news through technology platforms.

**Table 2:** Self-reported technology use of participants (n=1115).

Characteristics			Participants, n (%)
**Total technology use (h/d)**			
	<1	66 (5.9)	
	1-4	321 (28.8)	
	5-8	323 (29)	
	9-12	238 (21.4)	
	13-16	120 (10.8)	
	≥17	46 (4.1)	
**Change in technology use due to the COVID-19 pandemic**			
	Decreased or stayed the same	292 (26.2)	
	Increased by 1-2 h/d	275 (24.7)	
	Increased by 3-4 h/d	311 (28)	
	Increased by 5-6 h/d	128 (11.5)	
	Increased by ≥7 h/d	89 (8)	
**Comfortable using technology**			
	Not at all	204 (18.4)	
	Slightly	91 (8.2)	
	Moderately	187 (16.9)	
	Very	301 (27.1)	
	Extremely	326 (29.4)	
**Types of technology used during the COVID-19 pandemic**			
	Video chatting with friends or family (eg, Zoom, WhatsApp, and FaceTime)	902 (80.9)	
	Video meetings for work-related activities	614 (55.1)	
	Telephone or video visit with health care provider	575 (51.6)	
	Exercise or activities for physical fitness	420 (37.7)	
	Mental health activities	187 (16.8)	
	Keeping in touch with friends and family through social media	866 (77.7)	
	Leisure activities or hobbies	517 (46.4)	
	Accessing news	850 (76.2)	
	None	20 (1.8)	
	Other (please specify)	20 (1.8)	

**Figure 1:**
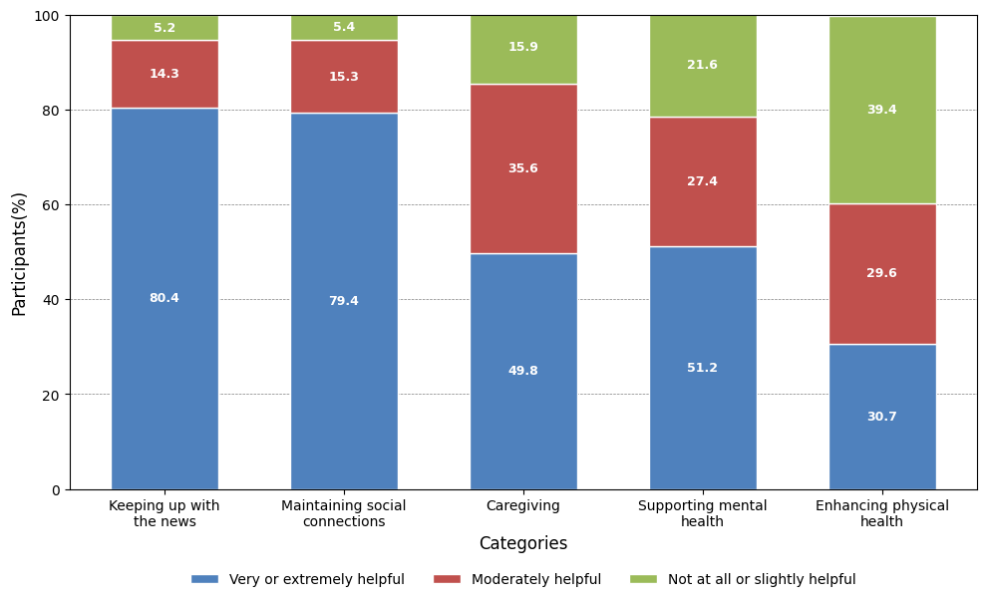
Survey responses to whether using technology was helpful in different aspects of life during the COVID-19 pandemic (n=1115).

The findings (Figure 1) indicate that the majority of participants (896/1115, 80.35%) found staying informed by accessing news through technology very or extremely helpful. In terms of keeping in touch with social connections, a significant majority (885/1115, 79.37%) perceived technology as very or extremely helpful, with only a small proportion (60/1115, 5.38%) reporting slightly or not at all helpful. Similarly, in the context of caregiving, a substantial portion of participants (938/1115, 84.12%) viewed technology as moderately or extremely helpful. However, perceptions regarding mental health support through technology varied, with a notable portion of participants (874/1115, 78.38%) finding it moderately or extremely helpful, while others (241/1115, 21.61%) indicated that technology was slightly or not helpful. Finally, participants’ perceptions of technology’s helpfulness for physical health varied, with a considerable proportion (565/1115, 50.67%) reporting technology being moderately or very helpful, while others (439/1115, 39.37%) expressed that technology was slightly or not helpful.

Technology use among participants revealed a spectrum of use patterns (Table 2). Notably, 36.23% (404/1115) of the participants reported using technology for ≥9 hours per day. Specifically, 5.91% (66/1115) of the participants used technology for <1 hour per day, while 28.78% (321/1115) used it for 1 to 4 hours per day, 21.34% (238/1115) for 5 to 8 hours per day, 21.34% (238/1115) for 9 to 12 hours per day, 10.76% (120/1115) for 13 to 16 hours per day, and 4.12% (46/1115) for ≥17 hours per day.

### Themes Related to Technology Use

Of the 5411 participants who completed the quantitative survey, 1115 (20.66%) provided responses to the open-ended question “Is there anything else you want to tell us about your use of technology during COVID-19?” A thematic analysis revealed four main themes related to digital technology use during the COVID-19 pandemic: (1) technology was critical for functioning across many aspects of life and maintaining physical, mental, and emotional well-being; (2) technology was often the only means of interpersonal social connections; (3) overuse led to negative physical and mental health outcomes; and (4) technology use was associated with multiple challenges and barriers.

#### Technology Was Critical for Functioning Across Many Aspects of Life and Maintaining Physical, Mental, and Emotional Well-Being

##### Overview

Technology use was found to be critical in maintaining daily activities related to skills and hobbies, work, and fitness during the pandemic. All these functions were reported to be vital for maintaining mental health, given the limited access to physical connection and ability to socialize outside of their homes. Participants reported feeling very isolated and believed that they would not have coped as well as they did without technology:

Without technology (internet, social networks, video streaming, online shopping), I’m not sure how I could survive this shelter-in-place.

Ethnic Chinese participant, male, aged 44 y

This reflects a common theme of isolation and disconnection experienced during the lockdown and shelter-in-place period. Many individuals were forced to physically distance from their community networks, resulting in increased reliance on technology to connect with others. In addition, many were unable to carry out routine errands, such as shopping, making delivery services their only option. One participant similarly emphasized her dependence on technology:

I would have gone absolutely insane without the internet during this quarantine.

Filipino participant, female, aged 40 y

During the lockdown, for many Asian American and Pacific Islander individuals, online access was the only means of connection to others. The internet was the safest way to follow public health guidelines, protect themselves and their loved ones from COVID-19 infection, and maintain their mental and emotional well-being. In terms of helpful aspects of technology, a participant described using social media to spread information and raise awareness on social issues relevant to Asian American and Pacific Islander individuals:

I used social media to spread information about anti-Asian discrimination and Black Lives Matter.

Vietnamese participant, female, aged 49 y

This statement reflects the broader context of the time, including the emotional unrest associated with the lockdown and the concurrent political movements and cultural propaganda directed toward the Asian American and Pacific Islander community. It highlights how these challenges were experienced by many individuals in this community and how they used social media as a tool for connection and raising awareness.

##### Skills and Hobbies

Many participants found technology to be beneficial for learning new skills and hobbies to pass time and for entertainment during the lockdown. These activities helped alleviate boredom and loneliness and created opportunities to explore new interests, for example, learning to cook, acquiring linguistic skills, and learning to play musical instruments. Such engagement was reported to help ease stress and remain occupied during the lockdown:

In my personal experience, the use of technology allowed me to learn and be exposed to people and places that may not have been possible. Also, I’ve become more knowledgeable with technology.

Filipino participant, female, aged 62 y

This quote illustrates how participants used technology to adapt and find new ways to connect with others as well as engage in new activities to promote positive habits and increase knowledge during the lockdown.

Another participant described how technology supported the continuation of daily activities and helped ease pandemic-related stress:

I have become better at the interplay between technology & non-technology- using technology to enhance aspects of my non-technology life like learning instruments or exercise.

Chinese participant, female, aged 47 y

This comment reinforces the theme of participants using technology to master new skills and hobbies to improve their quality of life during the shelter-in-place period.

##### Work

Learning new skills also included improving technology skills, such as adapting to online platforms for work and school. This included learning to navigate various tools and interfaces such as Zoom, Google Classroom, Google Workspace, Microsoft Office, and others:

I have had to learn to use a lot of technology tools for work within a very short period of time - Google classroom, Google slides, Google docs, Google drive, Ladibug document camera, Screencastify, Boom cards, interactive pdf, and of course, Zoom.

Chinese (Huaren) participant, female, aged 51 y

This quote reflects the transition to online forums and the use of new technological tools and how participants were required to quickly adapt to maintain productivity for work and school tasks during the lockdown.

Other benefits related to work included reduced commuting time and costs, which participants associated with increased efficiency and more time to focus on work-related tasks:

Good for my work I don’t have to commute everyday no Bart [train]/bus fares don’t have to wake up early to prepare going to work.

Filipino participant, female, aged 68 y

This supports the finding that new advances in technology and the incorporation of digital tools into work and school routines enabled participants to save time on commuting and increase productivity.

##### Fitness

Technology was reported to be especially helpful for monitoring and tracking fitness goals, particularly through devices such as Fitbit and Apple Watch:

I rely heavily on Fitbit to monitor and achieve physical fitness goals. This in turn has helped lower my blood pressure after these months of working out consistently.

Korean participant, female, aged 66 y

A Chinese participant (female, aged 60 y) shared that she was “doing virtual exercise classes daily,” and another participant highlighted the role of activity tracking:

Apple Watch (and other activity trackers) have been helpful to keep me moving.

Native Hawaiian participant, male, aged 25 y

These quotes reflect the usefulness of technology for achieving consistent physical activity and monitoring daily progress for many participants. Thus, technology helped participants meet their fitness goals and was a tool to support positive health outcomes.

#### Technology Was Often the Only Means of Interpersonal Social Connections

##### Overview

Technology helped address participants’ feelings of boredom and isolation by allowing access to streaming services, online shopping, and social media, as well as enabling them to feel connected to others. Staying connected through technology was also vital for work and health-related reasons, for example, when checking in on loved ones who were immunocompromised:

It’s been very important in keeping me sane and connected to others.

Vietnamese participant, female, aged 35 y

This quote supports the finding that technology was essential in maintaining social interaction during the pandemic.

##### Connections for Social Interactions

During the COVID-19–related lockdown, many participants were unable to physically check in with friends and family and had to rely on alternative methods to maintain social connection. Technology use was reported to ease negative feelings by allowing quick and effective communication. Many participants with family members and friends residing out of the country used platforms such as Facebook Messenger, WeChat, KakaoTalk, LINE, or WhatsApp for daily interactions and updates. Social media, Zoom, and Skype were also heavily used by participants to connect with loved ones and meet new people during this period of isolation. These tools were reported to be very useful for addressing communication challenges and easing worries of not being physically present:

Would have been much harder to cope without technology! Being able to video conference or text friends has helped maintain a sense of social connection while also being safe.

Chinese participant, female, aged 51 y

This quote further showcases technology’s ability to bridge gaps in social connection, enabling participants to use tools to interact with members of their communities and support one another while maintaining safe distancing practices.

Technology was also reported to help participants meet new people and find online groups centered around similar interests and hobbies:

I have been involved in decisions about moving my community chorus to the virtual platform, a less than satisfactory replacement to singing together. But we have been able to reach out to singers from far and wide, something we would not have been able to do before!

Chinese participant, female, aged 66 y

This quote illustrates how technology enabled participants to meet others with common interests and hobbies, further expanding their communities and social interactions. Another participant shared a similar experience:

It’s been an eye-opener to realize the capacity of the internet to connect people from all over the world through events, etc. I volunteer with our Girl Scout council and create virtual events for girls, who have attended from across the country!

Japanese participant, female, aged 67 y

This quote reflects the theme of technology facilitating the formation of new online communities, allowing participants to build relationships across geographic boundaries through common interests.

A participant expressed the importance of social connection with other Asian American and Pacific Islander individuals in the face of the racism and xenophobia that some experienced:

We Asians face a lot of discrimination. We can only communicate with friends online because it’s risky to go out, and technology supports this.

Chinese participant, female, aged 36 y

This quote reflects participant concerns regarding the hate directed at Asian American and Pacific Islander communities during the lockdown and illustrates how technology enabled safe social interaction and communication.

##### Connections to Help Others

Technology was also reported to assist in the care of others, such as ordering groceries and essential supplies through online platforms:

It was helpful that I could order groceries for myself and my elderly parents on Instacart and also I am able to Zoom with them a few times a week so that we can “see” each other make sure everything is ok.

Asian Indian participant, female, aged 53 y

The availability of no-contact services provided a practical alternative to in-person errands, helping participants reduce their risk of exposure to COVID-19 infection while continuing to care for loved ones.

##### Connections for Work

Participants reported benefits associated with the shift to remote work. One noted advantage was the ability to expand business contacts across geographic regions and use platforms such as Zoom to widen professional networks and communicate with people in different time zones.

##### Connections for Health Reasons

Participants reported that technology use also facilitated access to wellness resources and communication with health care professionals. This included using technology to connect with meditation groups, community meetings, workshops, online physical activity classes, and telehealth services. Technology also enabled video calls with loved ones in hospital when in-person visits were not allowed:

Use of technology in the hospital provides an effective source of communication between the patient and his/her family members. I witnessed how helpful and meaningful to keep in touch with our patients’ family by using smart phones or tablets, to see their loved one who is in the hospital in the screen really brought them a true happiness and brought positive effects to patients’ wellbeing.

Filipino participant, female, aged 52 y

This comment illustrates how technology was used to care for others and protect individuals considered vulnerable by maintaining safe distancing. It also reflects concern regarding disconnecting from loved ones during the lockdown and how using technology allowed participants to continue to provide care and support to their communities.

#### Overuse Led to Negative Physical and Mental Health Outcomes

A common theme among participants was that increased technology use during the lockdown led to overuse, contributing to negative mental and physical health outcomes, including burnout. Participants described how increased reliance on technology use led to the feeling that they needed a break from technology and having no outlets away from technology use, feelings of increased reliance, and addiction to technology and social media.

#### Negative Mental Health

Participants also described needing mental health breaks due to feeling overwhelmed by constant COVID-19 updates from news outlets and social media. Some reported feeling addicted to social media and finding it difficult to step away due to loneliness or fear of “missing out,” which resulted in increased anxiety and mental stress:

Technology, specifically social media has been incredibly stressful but addictive for me. I find myself much more anxious and unhappy when scrolling through other peoples profiles. But I feel this need to know otherwise I will miss out.

Vietnamese participant, female, aged 24 y

This quote reflects the broader theme of technology’s impact on mental health during the pandemic, highlighting how constant access to social media and news updates made it difficult to disconnect for fear of missing essential information.

Technology also enabled participants to stay informed about the pandemic by providing access to news updates from outside of their immediate communities, including other cities, states, and countries. However, this constant exposure to negative news was also associated with increased anxiety and stress:

It’s a double-edged sword—on one hand it has provided access to news and connection to family and friends, but it still feels isolating, lonely, and awkward to try and convey emotions and connections through the use of social media only.

Asian Indian participant, female, aged 24 y

This quote supports the finding that participants struggled to use technology effectively to maintain connection. Technology use helped participants stay connected with loved ones, but it also contributed at times to feelings of isolation and loneliness.

#### Discrimination Experienced by Asian American and Pacific Islander Individuals

Some participants reported negative experiences related to discrimination and racism during the pandemic, particularly on social media. Others expressed concerns about language barriers, xenophobia, and racism throughout this period:

I have been more sensitive to racially related news and posts on socially media.

Chinese participant, female, aged 56 y

[I] found that social media can be a bit more toxic during this period. Especially with all the negative news surrounding social justice, racism, the election, etc.

Chinese participant, male, aged 28 y

These quotes express the emotional and mental impact of frequent exposure to news and social media during a period marked by political unrest, social justice movements, and the pandemic, which resulted in participants feeling overwhelmed.

#### Burnout

Participants described feeling fatigued from constant technology use throughout the day but unable to disconnect due to the need to use technology for school and work:

I noticed that my technology usage has significantly increased during the COVID-19 pandemic. In doing so, I realized that I need to take screentime breaks so that I don’t get exhausted from it.

Filipino participant, female, aged 23 y

Participants reported exhaustion from prolonged use of digital devices and limited opportunities to disconnect, given the lack of in-person alternatives for connection with others during the pandemic. This led to participants incorporating breaks from technology to maintain their mental health.

Some individuals reported being busier during the pandemic due to increased work hours and workload. A participant shared her experience as a frontline worker:

During the pandemic I have never been busier. Following my shifts at the hospital, I attend nightly community, task force, and organizing meetings learning about resources and responses to the COVID-19 pandemic that is disproportionately affecting our community of immigrant front-line workers with high levels of high-risk exposure. Technology has made it easier to connect local and national Filipino organizations, but Zoom fatigue coupled with clinical burnout is a real hazard.

Filipino participant, female, aged 30 y

This quote illustrates how frontline workers experienced long shifts and overwhelming workloads to protect their patients.

Participants also reported experiencing Zoom fatigue and feeling exhausted from daily online meetings. Some also reported struggling to separate work from home after transitioning to fully remote work, leaving little downtime to destress:

I was working from home 4 days per week before the pandemic, so I was just on my computer all day every day already. Now I’m on it more because I can’t go out on weekends or in the evenings.

Chinese participant, female, aged 35 y

The combination of seemingly nonstop screen time from both work and personal activities contributed to mental and emotional exhaustion and resulted in feelings of burnout.

#### Negative Physical Health

Technology use during the pandemic was also found to be associated with negative physical health outcomes. Participants reported increased screen time and reduced physical activity, which contributed to symptoms such as fatigue, eyestrain, dry eyes, headaches, back and neck pain, wrist and hand pain, loss of sleep, dizziness, and weight gain:

Using electronic devices for long hours makes my shoulders, neck and back pains. However, I don’t feel safe to see a doctor or physical therapist.

Taiwanese participant, female, aged 37 y

Too much screen time, more sitting for work related issues, common illnesses experienced, like headache and back pain.

Asian Indian participant, female, aged 47 y

These narratives reflect the physical toll of sustained technology use that compounded over time during the pandemic. Notably, the hesitation to seek care due to safety concerns highlights how the pandemic exacerbated both physical strain and barriers to accessing care for technology-related health issues.

#### Technology Use Was Associated With Multiple Challenges and Barriers

Many participants shared concerns regarding increased technology use, including language-related communication challenges in virtual settings; increased costs; poor connectivity; and difficulty adapting to technology, particularly among older adults. These barriers may have been more pronounced for individuals who were underresourced or living in areas with limited internet access.

#### Challenges of Being a Nonnative English Speaker

Virtual work environments introduced new barriers for some Asian American and Pacific Islander individuals, as described by a participant:

As a nonnative English speaker/immigrant, I felt that I couldn’t perform as well during a Zoom job interview as I became very self-conscious about my accent or mistakes that I make when I speak English. I think the Zoom interview environment has negatively impacted my chance of landing a job because it encourages interviewers to rely more on interviewee’s speech as there are limited nonverbal cues that contributes to effective communication.

Korean participant, male, aged 33 y

This quote highlights the participant’s perceived disadvantages when navigating a virtual job interview, with interviewers unable to meet interviewees and assess them as a whole. With first impressions limited to screen interactions, the complete dependence on technology was a barrier to connection and, in this participant’s case, their success. Furthermore, relying primarily on speech disadvantaged the participant, a nonnative English speaker, heightening their fear of judgment and perceived risk of discrimination.

#### Increased Costs

Participants reported experiencing increased or additional expenses related to their increased technology use. These included upgrading internet service, paying higher data fees, and incurring increased electricity costs to support working or studying from home:

I had to upgrade my internet to be able to service people for work and during my internship for graduate school and it cost more to upgrade. Also, my electricity bill has gone up since I’m working and interning at home.

Japanese participant, female, aged 41 y

Increased costs posed additional challenges for individuals and families and was an added stressor, especially for those who were already facing financial strain.

#### Connectivity

Increased technology use at home also led to connectivity challenges related to school and work. Many participants reported that increasingly busy networks and limited bandwidth in certain areas led to decreased connectivity efficiency, which caused disruptions in work and school tasks. This often led to meeting disruptions, resulting in additional stress and loss of time to address connectivity issues:

With remote schooling and working remote, even in the suburbs, there are challenges with connectivity as everyone is online and using the internet for school and work. If the wireless networks and bandwidth are not strong, this often negatively impacts accessing internet for school and work.

Filipino participant, female, aged 47 y

This quote underscores that the usefulness of technology depends on reliable infrastructure. These challenges were especially pronounced for individuals in rural areas and for older adults who already faced barriers such as unfamiliarity with technology, financial constraints, and lack of support.

#### Technology Use Among Older Adults

Several participants reported difficulty navigating technology, with only 8.2% (91/1115) indicating that they felt slightly comfortable using technology and 18.4% (204/1115) reporting that they were not at all comfortable. The transition to increased technology use during the pandemic disproportionately affected older Asian American and Pacific Islander adults who struggled to adapt and often lacked the support necessary to learn how to operate technological tools. A participant expressed difficulty understanding how to use technology, stating that she would benefit from someone teaching her:

Please teach low tech adult/senior citizens like me how to use our iPhones, MacBook etc. We are dependent on our 7-12 year old grandchildren for tutorials (if we are lucky to have them nearby). Apple stores don’t teach us anymore.

Filipino participant, female, aged 77 y

This quote reflects the generational gap in technology familiarity and the desire of older adults to improve their technology literacy and decrease their dependence on younger family members in this regard. It also highlights the frustration that some older adults felt due to limited opportunities for formal instruction and underscores the need for structured, age-inclusive technology education tailored to older adults.

Some participants expressed concern that their older adult loved ones did not know how to use technology to contact resources or reach out to family members for assistance during the pandemic:

I am grateful that I am comfortable and experienced in all aspects of technology. However, my mother (who does not live with me) is not, and it always worries me that she wouldn’t be able to contact me if she has a pressing need, or that I am not around to help her immediately troubleshoot technological difficulties.

Taiwanese participant, female, aged 31 y

This participant’s sentiment underscores the stress that families experienced when older adults lacked the skills to use technology independently. Limited technology access can create safety and communication barriers, especially during emergencies and periods of physical separation.

Another participant similarly reported worries regarding an older loved one:

Using technology is difficult with my elderly mom as she does not understand how to use it. As she is in an assisted living home, I cannot show her and the staff has limited time to help. For the hearing impaired or for those who are not familiar with screen interactions, technology is limited in its usefulness for meeting social needs.

Chinese participant, female, aged 50 y

This quote encapsulates the compounded challenges that older adults face when navigating technology, even in assisted living settings, and highlights how barriers such as impairments or unfamiliarity with digital interfaces can significantly hinder social connectivity and access to essential support.

## Discussion

### Principal Findings

This study describes the use of technology and its impact on the well-being and experiences of Asian American and Pacific Islander adults during the COVID-19 pandemic. While other studies have investigated the broad implications of technology on mental and physical health, the use of technology and its application to the culturally specific experiences of Asian American and Pacific Islander adults during the pandemic was largely unknown. The quantitative survey data revealed significant changes in lifestyle and communication behaviors that occurred during the pandemic. Nearly half of the respondents (528/1115, 47.5%) reported increasing their technology use by ≥3 hours per day during the pandemic, with up to 7.98% (89/1115) indicating an increase of ≥7 hours per day compared to before the pandemic. The 4 themes identified in this study highlight the mixed sentiment toward technology and social media use among Asian American and Pacific Islander adults, alongside notable challenges and barriers.

In the periods of social isolation during the pandemic, technology was a central force in facilitating individual well-being and fostering connections within the community [16]. Virtual platforms enhanced both the professional and hobby-related skills of participants, while connecting individuals with common interests. In addition to supporting mental and emotional health, technology benefited the physical health of participants through the use of fitness trackers and virtual interactions with health care professionals. Moreover, for the Asian American and Pacific Islander community, technology assumed a special significance by providing a sense of security amid rising anti-Asian discrimination. Virtual platforms and networks helped individuals stay informed about relevant social issues and incidents affecting their communities. Fearing for their safety, Asian American and Pacific Islander adults relied on technology, such as video chats and telephone calls, to maintain social connections without leaving home. Older adults were also better supported by family members who used mobile delivery services to order food and other essentials.

However, alongside the benefits of using technology, the detrimental effects of overuse on both mental and physical health were also revealed by this study. Participants reported concerns such as addiction to social media, burnout, and videoconferencing fatigue, which exacerbated feelings of loneliness and mental strain. For Asian American and Pacific Islander adults in particular, the increased exposure to negative news and discrimination on social media contributed to heightened awareness of discrimination, which in turn led to psychological distress. In addition to these negative impacts on mental health, technology overuse was associated with physical symptoms such as fatigue, eyestrain, back pain, headaches, and weight gain.

Our analysis of technology-related barriers for Asian American and Pacific Islander adults underscored how technology may exacerbate existing disparities within the community. Notably, technology may amplify inequities in employment opportunities, particularly evident in the challenges faced by nonnative English speakers during job interviews conducted over platforms such as Zoom. The shift to virtual interviews accentuated concerns about linguistic proficiency. Asian American and Pacific Islander participants expressed concern that interviewers may prioritize verbal communication over nonverbal cues, placing nonnative English speakers at a disadvantage. Moreover, increased reliance on technology also imposed financial burdens, potentially compounding economic disparities within the community. In addition, the growing global reliance on technology presented a significant barrier for older adults in the Asian American and Pacific Islander community who struggle to learn new technology. This challenge had a ripple effect on family members of these older adults, as they faced increased distress fearing an inability to contact older adult members in emergent situations or in times of social isolation.

While the themes of our study reveal a spectrum of positive and negative impacts of technology on Asian American and Pacific Islander adults, such mixed results regarding technology use are congruent with current literature sampling the general population. Our finding that technology was critical in maintaining individual mental and emotional well-being during the pandemic is supported by both studies concluding that the use of technology and digital tools was beneficial for young adults during the COVID-19 pandemic [17] and studies involving older adults [18,19]. In alignment with our findings, these studies reported reduced feelings of loneliness, better self-rated health, fewer chronic illnesses, higher subjective well-being, and fewer depressive symptoms. The use of technology as a facilitator for the feeling of community connection has also been well reported. A qualitative study involving older adults and their relatives highlighted how the use of technology-mediated communication by older adults during the pandemic afforded meaningful interconnections in their social lives and promoted cross-generational connections among family members [20].

Our finding that technology overuse can lead to detrimental effects on the mental and physical health of Asian American and Pacific Islander adults is also congruent with prior literature. Prior research has shown that moderate or severe depression levels were associated with greater time spent watching television and using computers (>6 h/d) for American adults [21]. In addition, our parent study of Asian American and Pacific Islander individuals found that those whose technology use during the pandemic increased by ≥7 hours per day had a 1.32-point (95% CI 1.00-1.64; *P* value for the trend <.001) higher Patient Health Questionnaire-4 score [9]. Negative impacts on physical health in the Asian American and Pacific Islander population are also congruent with our previous study results, which detected significant changes in self-reported physical health scores with ≥3 hours per day of technology use [9], as well as independent studies reporting that technology use negatively affects physical activity and sleep routines, leading to headaches, neck pain, myopia, digital eye syndrome, and cardiovascular risk factors [22,23].

Finally, and perhaps most notably, our fourth theme, which discusses the multiple challenges and barriers to technology use for Asian American and Pacific Islander adults, contributes to the highly necessary investigation of racial disparities in an increasingly digitized world. Nonnative English speakers in this study described feeling disadvantaged in virtual workspaces, an experience recognized by a growing number of studies. In a case study of a multinational corporation after a shift to remote work triggered by the COVID-19 pandemic, researchers found that language-based discrimination in virtual spaces was primarily organizational and more subtle than in physical settings [3]. Virtual spaces also seemed to expedite the process of excluding migrant professionals, in part due to the emergence of separate meetings and parallel virtual channels for informal conversation [3]. Other barriers observed in our study, such as increased costs, connectivity challenges, and the lack of technological support for older adults, add to the growing body of evidence that technology may be exacerbating, rather than eliminating, socioeconomic and health disparities through the digital divide. This divide has significant clinical implications for the Asian American and Pacific Islander community.

This study illustrates ways in which Asian American and Pacific Islander adults found technology to be beneficial for mental and physical health. These results contradict our prior study results, which indicated that an increase in technology use during the pandemic correlated with negative mental health outcomes in the form of higher Patient Health Questionnaire-4 scores and lower physical health scores [6]. This discrepancy may be due to the variance in the framing of questions between the two studies. Our prior study (COMPASS) analyzed numeric responses from a structured set of questions, while this study analyzed responses to the open-ended question “Is there anything else you want to tell us about your use of technology during COVID-19?” This may have permitted respondents to consider alternative, positive applications of technology, such as its use as a tool to safeguard against an anti–Asian American and Pacific Islander social environment.

As revealed by our findings, while the COVID-19 pandemic accelerated the use of technology and telehealth, it also exacerbated existing challenges and barriers to accessing and using needed health care, evident in the growing digital divide and lack of digital literacy, as discussed in prior literature [24-26]. Asian American and Pacific Islander adults, particularly those with limited English proficiency, older adults, individuals with low income, and those living in rural areas or neighborhoods experiencing internet connectivity issues or lacking necessary digital literacy skills to effectively use telehealth platforms, face more challenges in adopting and using telehealth [27,28]. This leads to increased health disparities due to inequitable access to necessary health care. The barriers observed in our study, such as poor digital literacy, internet access issues, and lack of technology assistance, have been proven to be key drivers of the digital divide [29]. As such, identifying and resolving these barriers within the Asian American and Pacific Islander population is vital for the advancement of equitable health care and for appropriately addressing technology use as a social determinant of health. While many members of Asian American and Pacific Islander communities have become significantly more aware of the need to use such technologies, it is both crucial and timely to provide culturally and linguistically appropriate services, as well as training and resources to improve digital literacy, to address the identified disparities.

Consistent with previous research [30], our findings have revealed that dependence on technology, such as increased screen time, was associated with negative mental health. Conversely, avoidance of technology due to anxiety or lack of familiarity can prevent individuals from accessing telehealth services, potentially leading to delayed treatment or untreated health issues. Clinicians should be aware of these potential issues and integrate assessments of technology use and its impact on mental health into their care plans. Using a patient-centered approach to provide the needed support and resources to manage technology use in a helpful manner and offering alternative care options for patients who prefer not to use technology are important strategies in addressing these concerns.

The digital divide—highlighted during the COVID-19 pandemic as a disparity between the “haves” and “have-nots”—has been well documented [31]; however, more research is required to better understand the needs of diverse populations, not only by race and ethnicity, but also by English proficiency and age. This study contributes important knowledge in this area and may be helpful for developing social and educational programs to serve such diverse populations (eg, Asian American and Pacific Islander adults with low socioeconomic status and limited English proficiency).

### Limitations

This study has several limitations. We used convenience sampling through community networks, social media, and the CARE registry, which may not fully capture the diversity of the broader Asian American and Pacific Islander population. There may be selection bias, with underrepresentation of individuals with limited internet access or those not engaged with community organizations. In addition, only 20.66% (1115/5398) of the respondents to the quantitative survey provided open-ended responses, which formed the basis for the qualitative themes presented in this study. In terms of generalizability, although the age range of participants was 18 to 98 years, the mean age was 45.9 (SD 16.3) years, which may reflect a generally more technology-savvy group. Furthermore, the majority of the respondents (665/1115, 59.64%) completed the open-ended question in English, likely reflecting a sample with a higher level of acculturation to the United States. Finally, we reported aggregated data across numerous Asian American and Pacific Islander groups, which may not represent the unique experiences of specific backgrounds within these communities.

### Conclusions

This study presented the diverse attitudes and experiences related to technology use among Asian American and Pacific Islander adults during the COVID-19 pandemic. There were numerous benefits of using technology during a period of mandated physical and social isolation; however, there were many adverse experiences, including the detrimental effects of technology overuse on both mental and physical health. Important considerations for Asian American and Pacific Islander adults include awareness of how technology may exacerbate existing disparities, particularly among those with limited English proficiency. Further qualitative research would be beneficial in amplifying the perspectives of Asian American and Pacific Islander adults to uncover concerns and address health disparities.

## Appendix

Multimedia Appendix 1 Technology survey questions.

## Abbreviations

CARE: Collaborative Approach for Asian Americans and Pacific Islanders Research and Education
COMPASS: COVID-19 Effects on the Mental and Physical Health of Asian Americans and Pacific Islanders Survey Study
UCSF: University of California San Francisco

## Data Availability

The datasets generated or analyzed during this study are available from the corresponding author on reasonable request.
