# Supplementary material for: Diverse Attitudes and Experiences With Technology Use During the COVID-19 Pandemic Among Asian American and Pacific Islander Adults (the COMPASS Study): Survey Study
Source: JMIR Hum Factors. 2025 Sep 10;12:e64999. doi: 10.2196/64999 (PMC12461168; doi:10.2196/64999)
Supplement: Multimedia Appendix 1 [file humanfactors_v12i1e64999_app1.docx]

**Technology Survey Questions**

**Now we would like to ask you about your use of technology during the COVID-19 pandemic.**

- 1. **Do you own or have easy access to (check all that apply):**
- A smartphone
- Desktop or laptop computer
- Home internet (Wi-Fi)
- Tablet (iPad, e-reader, etc.)
- Television with cable
- TV streaming device (Roku, Apple TV, Fire Stick, etc.)
- None
- Other (please specify): ____________
  1. **As a result of the COVID-19 pandemic, I used technology for (check all that apply):**
- Video chatting with friends or family (Zoom, WhatsApp, FaceTime, etc.)
- Video meetings for work-related activities
- Phone or video visit with a health care provider
- Exercise or activities for physical fitness
- Mental health activities
- Keeping in touch with friends and family through social media
- Leisure activities or hobbies
- Accessing news
- None
- Other (please specify): ____________
  1. **How many hours are you connected to technology during the day?**
- <1 hour/day
- 1-4 hours/day
- 5-8 hours/day
- 9-12 hours/day
- 13-16 hours/day
- over 17+ hours/day
  1. **Did your use of technology increase during the COVID-19 pandemic?**
- Decreased
- Stayed the same
- Increased by 1-2 hours/day
- Increased by 3-4 hours/day
- Increased by 5-6 hours/day
- Increased by 7+ hours/day
- Unsure

**Please state your level of agreement with each of these sentences:**

- 1. **I am comfortable with using technology and do not usually experience difficulties.**
- Not at all
- Slightly
- Moderately
- Very
- Extremely
  1. **Using technology is helpful for my physical health:**
- Not at all
- Slightly
- Moderately
- Very
- Extremely
  1. **Using technology is helpful for my mental health:**
- Not at all
- Slightly
- Moderately
- Very
- Extremely
  1. **Using technology is helpful for keeping up with the news:**
- Not at all
- Slightly
- Moderately
- Very
- Extremely
  1. **Using technology is helpful for keeping in touch for social connections:**
- Not at all
- Slightly
- Moderately
- Very
- Extremely
  1. **Using technology is helpful to care for others:**
- Not at all
- Slightly
- Moderately
- Very
- Extremely
  1. **Is there anything else you want to tell us about your use of technology during the COVID-19 pandemic?**

________________________________________________________________________

________________________________________________________________________

________________________________________________________________________
